# Supplementary material for: Estimating treatment costs for uncomplicated diabetes at a hospital serving refugees in Kenya
Source: PLoS One. 2022 Oct 26;17(10):e0276702. doi: 10.1371/journal.pone.0276702 (PMC9604983; doi:10.1371/journal.pone.0276702)
Supplement: S1 Table — (DOCX) [file pone.0276702.s001.docx]

## **S1 Table. Methodology, data requirements, calculations, and sources of data.**

| **Health service activity** | **Cost item** | **Methodology** | **Data requirements** | **Calculation** | **Source** |
| --- | --- | --- | --- | --- | --- |
| **Outpatient consultation** | Staff | Top-down micro-costing | Number and staff types in OPD  Annual staff salary and fringe benefits  Productive work hours for each staff type in a year  Hours spent by each staff type for diabetic consultations in OPD in 2017  Total number of diabetic consultations in OPD in 2017 | Total annual staff cost = Annual salary + Annual fringe benefits  Staff cost per hour = Annual staff cost / productive work hours in a year  **Annual staff cost allocated to diabetes consultations in OPD in 2017** = Hours spent for diabetic consultations in OPD in 2017 x staff cost per hour  **Staff cost per diabetes consultation in OPD in 2017** = Annual staff cost allocated to diabetes consultations in OPD in 2017/ Total number of diabetic consultations in OPD in 2017 | HR records  Staff interviews  Staff activity log forms  Time and motion studies  Health Information System (HIS) records |
|  | Building | Top-down micro-costing | Surface area in square meters occupied by OPD rooms  Total surface area of the building in which OPD is located  Cost of putting up a similar new building as the building in which OPD is located  Useful life years of the building  Interest rate  % of time the space is used for diabetic consultations versus all other consultations conducted in OPD as it is a shared space with other clinics  Total number of diabetic consultations in OPD in 2017 | % allocation of space to OPD = surface area of OPD rooms / Total surface area of the building in which OPD is located  Use useful years and interest rate to obtain annualization factor  Total adjusted annual OPD building cost = (% allocation of space to OPD x Building cost of building in which OPD is located) / annualization factor  **Annual building cost allocated to diabetes consultations in OPD in 2017** = Total adjusted annual OPD building cost x % of time the space is used for diabetic consultations versus all other consultations  **Building cost per diabetes consultation in OPD in 2017** = Annual building cost allocated to diabetes consultations in OPD in 2017 / Total number of diabetic consultations in OPD in 2017 | Building surface areas were measured using a digital tape measure  Supply chain department records  Staff interviews  Staff activity log forms  Time and motion studies  Central Bank of Kenya statistics  WHO-CHOICE project |
|  | General equipment & furniture | Top-down micro-costing | Number and types of general equipment and furniture in the OPD consultation rooms  Unit replacement costs of all identified equipment and furniture  Useful life years  Interest rate  Total number of diabetic consultations in OPD in 2017  Total number of all consultations in OPD in 2017 | Total replacement cost of equipment and furniture = Unit replacement cost x quantity  Use useful years and interest rate to obtain annualization factor  Total adjusted annual equipment and furniture cost = Total replacement cost / annualization factor  **Annual cost of equipment & furniture allocated to diabetes consultations in OPD in 2017** = Total adjusted annual equipment and furniture cost x (Total number of diabetic consultations in OPD in 2017/ Total number of consultations in OPD in 2017)  **Equipment and furniture cost per diabetes consultation in OPD** in 2017 = Annual cost of equipment & furniture allocated to diabetes consultations in OPD in 2017 / Total number of diabetic consultations in OPD in 2017 | Supply chain department records  Central Bank of Kenya statistics  WHO-CHOICE project  Health Information System (HIS) records |
|  | Overhead | Top-down gross costing | Total annual direct (health program) costs incurred by the Dadaab site  Total annual indirect costs incurred by the Dadaab site. This included local administration costs, head office (Kenya) and head quarter (New York) administration costs allocated to the Dadaab site, general building cost for space occupied by general administration, general equipment cost for equipment used by general administration and vehicle cost | Sum up all direct costs  Sum up all annual indirect cost after inflation adjustment (if applicable)  **Mark-up percentage** = Total annual direct cost / Total annual indirect cost  **Overhead cost** = Total annual disease specific direct costs x mark-up percentage | Staff interviews  Financial data  Supply chain department records  Central Bank of Kenya statistics  WHO-CHOICE project |
| **Laboratory investigation** | Staff | Top-down micro-costing | Number and staff types in the laboratory department  Annual staff salary and fringe benefits  Productive work hours for each staff type in a year  Minutes spent per test for an RBS test  Number of RBS tests conducted in 2017  Number of all tests conducted by the lab in 2017 | Total annual staff cost = Annual salary + Annual fringe benefits  Proportion of staff time spent on RBS tests in 2017 = Hours spent on all RBS tests in 2017 / Total productive work hours of lab staff in a year  **Annual staff cost for RBS tests** = Total annual staff cost x Proportion of staff time spent on RBS tests in 2017  **Annual staff cost per RBS test** = Annual staff cost for RBS tests / Number of RBS tests conducted in 2017  Repeat process for HbA_1c_ tests | HR records  Staff interviews  Staff activity log forms  Time and motion studies  Lab registers |
|  | Test consumables | Bottom-up micro-costing | List of test consumables, quantities used in an RBS test and unit price of each consumable  Number of RBS tests conducted in 2017 | **Cost of consumables per RBS test** = Unit cost x quantity used in an RBS test x % allocation of consumable to an RBS test  **Total annual cost of consumables RBS tests in 2017** = Cost of consumables per test x number of RBS tests conducted in 2017  Process repeated for HbA_1c_ tests | Staff interviews  Supply chain department records  Lab registers and records |
|  | Medical equipment | Top-down micro-costing | List of medical equipment used in an HbA_1c_ test  Number of each of the identified equipment in the lab  Unit replacement cost of each equipment  Useful life years  Interest rate  Proportion of time the equipment is used for HbA_1c_ tests  Number of HbA_1c_ tests conducted in 2017  Number of all tests conducted by the lab in 2017 | Total replacement cost of medical equipment = Unit replacement cost x quantity  Use useful years and interest rate to obtain annualization factor  Total adjusted annual medical equipment cost = Total replacement cost / annualization factor  **Total adjusted annual cost of medical equipment used HbA_1c_ tests** = Total adjusted annual medical equipment cost x Proportion of time the equipment is used for HbA_1c_ tests  **Medical equipment cost per HbA_1c_ tests** = Total adjusted annual cost of medical equipment / Number of HbA_1c_ tests conducted in 2017  Process is not repeated for RBS tests because there was no medical equipment used in RBS tests by definition of equipment used in this study | Staff interviews  Supply chain department records  Lab registers and records  Supplier interview  Central Bank of Kenya statistics |
|  | Building | Top-down micro-costing | Surface area in square meters occupied by the lab  Total surface area of the building in which the lab is located  Cost of putting up a similar new building as the building in which the lab is located  Useful life years of the building  Interest rate  Number of RBS tests conducted in 2017  Number of all tests conducted by the lab in 2017 | % allocation of space to lab = surface area of lab / Total surface area of the building in which the lab is located  Use useful years and interest rate to obtain annualization factor  % allocation of space to lab = Surface area of lab / Surface area of building in which lab is located  Proportion of RBS tests to all tests conducted by the lab in 2017 = Number of RBS tests conducted in 2017 / Number of all tests conducted by the lab in 2017  Total adjusted annual lab building cost = (% allocation of space to lab x Building cost of building in which lab is located) / annualization factor  **Annual building cost allocated to RBS tests in 2017** = Total adjusted annual lab building cost x Proportion of RBS tests to all tests conducted by the lab in 2017  **Building cost per RBS test in 2017** = Annual building cost allocated to RBS tests in 2017) / Number of RBS tests in 2017  Process repeated for HbA_1c_ tests | Building surface areas were measured using a digital tape measure  Supply chain department records  Staff interviews  Staff activity log forms  Time and motion studies  Central Bank of Kenya statistics  Laboratory registers  WHO-CHOICE project |
|  | General equipment and furniture | Top-down micro-costing | Number and types of general equipment and furniture in the lab  Unit replacement costs of all identified equipment and furniture  Useful life years  Interest rate  Proportion of staff time spent on RBS tests | Total replacement cost of equipment and furniture = Unit replacement cost x quantity  Use useful years and interest rate to obtain annualization factor  Total adjusted annual equipment and furniture cost = Total replacement cost / annualization factor  **Annual cost of equipment and furniture allocated to RBS tests in 2017** = Total adjusted annual equipment and furniture cost x proportion of staff time spent on RBS tests  **General equipment and furniture cost per RBS test in 2017** = Annual cost of equipment and furniture allocated to RBS tests in 2017 / Total number of RBS tests conducted in 2017  Process repeated for HbA_1c_ tests | Staff interviews  Staff activity log forms  Time and motion studies  Supply chain department records  Central Bank of Kenya statistics  WHO-CHOICE project  Laboratory registers |
|  | Overhead | Top-down gross costing | Total annual direct (health program) costs incurred by the Dadaab site  Total annual indirect costs incurred by the Dadaab site. This included local administration costs, head office (Kenya) and head quarter (New York) administration costs allocated to the Dadaab site, general building cost for space occupied by general administration, general equipment cost for equipment used by general administration and vehicle cost | Sum up all direct costs  Sum up all annual indirect cost after inflation adjustment (if applicable)  **Mark-up percentage** = Total annual direct cost / Total annual indirect cost  **Overhead cost** = Total annual disease specific direct costs x mark-up percentage  Process repeated for HbA_1c_ tests | Staff interviews  Financial data  Supply chain department records  Central Bank of Kenya statistics  WHO-CHOICE project |
| **Drug treatment (monthly and annual regimen)** | Type 1 diabetes | Bottom-up micro-costing | List of drugs and consumables used to treat Type 1 diabetes  Dosage of each drug  Quantity of consumables used to deliver dosage  Unit price of each drug and consumable | **Total cost of drug regimen per patient per year** = Quantity of drug/consumable used in a year x Unit price  **Monthly cost of drug regimen per patient** = Total cost of drug regimen per patient per year / 12 | Staff interviews  Pharmacy registers |
|  | Type 2 diabetes | Bottom-up micro-costing | List of drugs used to treat type 2 diabetes  Dosage of each drug  Unit price of each drug | **Total cost of drug regimen per patient per year** = Quantity of drugs used in a year x Unit price  **Monthly cost of drug regimen per patient** = Total cost of drug regimen per patient per year / 12 | Staff interviews  Pharmacy registers |
