# Supplementary material for: Estimating treatment costs for uncomplicated diabetes at a hospital serving refugees in Kenya
Source: PLoS One. 2022 Oct 26;17(10):e0276702. doi: 10.1371/journal.pone.0276702 (PMC9604983; doi:10.1371/journal.pone.0276702)
Supplement: S2 Table — (DOCX) [file pone.0276702.s002.docx]

## **S2 Table. Distribution of economic costs**

| **Cost of health service activities in diabetes care^a^** | | | | |  |
| --- | --- | --- | --- | --- | --- |
| **Activity** | | **Output** | **Unit cost** | **Annual cost** |  |
| **OPD** | | **Visits** |  | |  |
| Consultation (total) | | 3,140 | 2.58 | *8,099.94* |  |
| Staff | | 3,140 | 1.38 | 4,319.54 |  |
| Building | | 3,140 | 0.28 | 883.66 |  |
| Equipment & furniture | | 3,140 | 0.01 | 44.85 |  |
| Overhead | | 3,140 | 0.91 | 2,851.89 |  |
| **Laboratory** | | **Tests** |  | |  |
| RBS test (total) | | 9,512 | 1.37 | 13,098.72 |  |
| Staff | | 9,512 | 0.39 | 3,751.94 |  |
| Test consumables | | 9,512 | 0.47 | 4,435.59 |  |
| Building | | 9,512 | 0.03 | 279.60 |  |
| Equipment & furniture | | 9,512 | 0 | 19.69 |  |
| Overhead | | 9,512 | 0.48 | 4,611.90 |  |
| HbA_1c_ test (total) | | 90 | 14.84 | 1,335.57 |  |
| Staff | | 90 | 3.14 | 283.04 |  |
| Test consumables | | 90 | 4.17 | 374.97 |  |
| Medical equipment | | 90 | 2.26 | 203.18 |  |
| Building | | 90 | 0.03 | 2.65 |  |
| Equipment & furniture | | 90 | 0.02 | 1.49 |  |
| Overhead | | 90 | 5.22 | 470.24 |  |
| **Cost Of Drug Treatment^a^** | | | | |  |
|  | | **Dosage period** | **Monthly cost** | **Annual cost** |  |
| T1DM regimen | | 365 days | 7.66 | 91.93 |  |
| T2DM regimen | | 365 days | 1.69 | 20.34 |  |
| **UNIT COST OF HEALTH SERVICE ACTIVITIES BY DIABETES TYPE^a^** | | | | |  |
|  | ***T1DM***  ***(RBS test)*** | ***T1DM***  ***(HbA_1c_ test)*** | ***T2DM***  ***(RBS test)*** | ***T2DM***  ***(HbA_1c_ test)*** |  |
| OPD consultation | $2.58 | $2.58 | $2.58 | $2.58 |  |
| Test | $1.59 | $14.84 | $1.59 | $14.84 |  |
| Drug treatment | $7.66 | $7.66 | $1.69 | $1.69 |  |
| Total cost | $11.83 | $25.08 | $5.86 | $19.11 |  |
| ^a^All costs were converted to USD (KES 103.37 = USD 1) using the average exchange rate for 2017 – adapted from The World Bank. Official exchange rate (LCU per US$, period average) - Kenya \| Data. Published 2017. <https://data.worldbank.org/indicator/PA.NUS.FCRF?locations=KE> | | | | | |
