# Supplementary material for: Estimating treatment costs for uncomplicated diabetes at a hospital serving refugees in Kenya
Source: PLoS One. 2022 Oct 26;17(10):e0276702. doi: 10.1371/journal.pone.0276702 (PMC9604983; doi:10.1371/journal.pone.0276702)
Supplement: Attachment — Submitted filename: Response to Reviewers.docx [file pone.0276702.s003.docx]

PONE-D-22-03317

**Estimating treatment costs for uncomplicated diabetes at the International Rescue Committee Hagadera Hospital in Kenya**

Dear reviewers,

Thank you very much for reading and reviewing our manuscript. We appreciate your objective feedback which has helped improve the manuscript substantially. Please see below for itemised responses to each of your comments.

Yours sincerely,

Dr. Lucy Kanya.

Reviewer #1: • General comments

This paper addresses a relevant topic, that of diabetes, and in a relevant population, that of refugees. It is well written and the methods appear rigorous enough, from what I can judge, since I am not an economist. The results are probably useful for international NGOs providing medical care to refugees. However, one can question its usefulness for other stakeholders; the authors will want to expand on this in their discussion and perhaps also in their conclusion.

Thank you for your comments. We have expanded on the usefulness of these findings in the discussion section and conclusion.

• Specific comments

1. In the title, is it possible to use “hospital serving refugees in Kenya” instead of giving the name of the hospital?

Thank you. Title adjustment made to “hospital serving refugees in Kenya”.

2. Abstract: RBS should be defined.

Random blood sugar (RBS) tests are now defined in the abstract.

3. Why refer to “holistic” management when there is no consideration of prevention or follow-up in the paper?

Our study methodology has taken into account the cost of follow-up for patients with diabetes. However, given the humanitarian context and its associated data collection challenges, we were unable to differentiate among newly diagnosed patients and those who came for a re-visit. Nevertheless, the treatment protocols followed within the facility were similar for both patients segments. However, we do acknowledge that prevention costs are not included. Holistic management in our analysis therefore refers to the care that patients receive from the point of testing, and subsequent care when the patients return to the facility for a revisit. We have included this in the revised manuscript.

4. It would be useful for the readership to have an idea of the estimated prevalence of diabetes in the refugee camp.

It is estimated that the Hagadera refugee camp has nearly 1,000 patients with diabetes under follow-up in 2018. In 2017, diabetes patients represented 11.76% of the OPD consultations. However, no previous diabetes prevalence studies have been conducted in the camp. The manuscript has been updated.

5. In Africa, diabetes is usually diagnosed once there are already complications unless active screening takes place. Of the patients treated in 2017, how many did NOT have complications? Also, is information on the time since diagnosis available?

This observation is correct and is likely the case in the study setting. However, diabetes complications were not captured as they were out of the scope of this paper. In addition, the study has focused on refugees; excluding the general population in Kenya.

6. This study took place before the pandemic: this is perhaps an important point to mention.

Thank you. This has now been included in the paper in the limitations section.

7. If I understood correctly, it is assumed that patients with type 2 diabetes are solely on hypoglycemic drugs, not on insulin? Please explain.

Unfortunately, access to insulin is very challenging within humanitarian settings (and the general population in Kenya) and it is often reserved for those with type 1 diabetes.

8. The content of the interviews should be briefly described.

Interviews were conducted to determine inputs required for diabetes care, and to estimate staff time allocation. The manuscript is updated.

9. It would be important to know why the health posts were not included in the study and to include this as a limitation of the study.

Thank you for the suggestion to include this as a limitation of our study. The incurred costs at the health posts were out of scope of the study as these services are provided by community health workers (CHWs) who also provide other services including maternal and child health, infectious diseases etc. Consequently, it was difficult to collect data specific to diabetes follow ups at the community level. We mention this in the manuscript too.

10. Do the patients pay for consultations, tests or drugs? If everything is free for the refugees, then the findings are not applicable where patients have to pay out of their pocket, which is the case in most African countries.

Thank you for your question. The study has followed a provider perspective approach when calculating the estimated costs. Hence, out of pocket (OOP) and other societal costs were outside the scope of the study. Such costs are likely significant in a country like Kenya where OOP health expenditure is estimated at more than one-fifth (as stated in the limitations section), however, it is important to note that there was no expenses for patients to receive services at the hospital. The analysis from a provider perspective are therefore very important for program implementers, including payers such as the government of Kenya in their plans to extend universal health care coverage to its populace.

11. Is it possible to explain the term “gross-costings”, and “top-down” and “bottom-up” micro-costing, for non-economists?

Noted. This has now been included in the manuscript.

12. This costing method may be of value for other NCDs; the authors will want to say a word on this.

Thank you for raising this important point. We have included a statement within the conclusion demonstrating that similar costing methods would be useful for other NCDs, with tools adapted according to treatment pathways for each NCD.

Reviewer #2: I read your paper with great interest. The topic is novel. It is better to improve the English language of the paper. There are several mistakes. Moreover, it is better to use clear language with small sentences.

Studied----study

Which---the which

As the evidence----as evidence

Which accounted----that accounted

and direct/indirect cost items --- and direct/indirect cost items are

The clinical pathway for care of uncomplicated diabetes------ The clinical pathway for the care of uncomplicated diabetes

Measurement, valuation and allocation of costs to health----- Measurement, valuation, and allocation of costs to the health

aAdapted----adapted

useful---of useful

organisational----organizational

Cost analyses contributes critically into economic evaluations.------- Cost

analyses contribute critically to economic evaluations.

Analysed----analyzed

At 2008---of 2008

İn direct costs-----indirect costs

Such---such as

World-wide----worldwide

Thank you very much for your feedback. We have revised the manuscript accordingly and maintained consistency in the use of English (UK).

Are the costs in your study annual or not? Please mention.

Costs are reported as unit costs. Kindly refer to table 3 for the estimated unit costs in USD. Additional details were also added in table 1 for the annualization factors that were used where applicable.

The discussion part of the paper should be improved. Please add more references. Please compare them. Please read these papers and cite them.

1. S.Ö. Keskek, S. Kırım, N. Yanmaz, N. Sahinoglu Keskek, G. Ortoglu, A. Canataroglu , "Direct medical cost of type 1 and type 2 diabetes in Turkey", International Journal of Diabetes in Developing Countries, Ekim-2013, DOI 10.1007/s13410-013-0159-6

2. Ş.Ö. Keşkek, S.Kırım, N. Yanmaz. Estimated costs of the treatment of diabetic foot ulcers in a tertiary hospital in Turkey. Pak J Med Sci 2014; 30(5): 968-971. doi: <http://dx.doi.org/10.12669/pjms.305.5182>

3.Gülümsek E, Keşkek ŞÖ. Direct medical cost of nephropathy in patients with type 2 diabetes. Int Urol Nephrol. 2021 Oct 18. doi: 10.1007/s11255-021-03012-

Thank you very much for sharing these references. While they are not specifically focussed on a humanitarian setting as our analysis is, we have read and included findings and conclusions from them in the discussion section of the revised manuscript.

6. PLOS authors have the option to publish the peer review history of their article ([what does this mean?](https://journals.plos.org/plosone/s/editorial-and-peer-review-process#loc-peer-review-history)). If published, this will include your full peer review and any attached files.

If you choose “no”, your identity will remain anonymous but your review may still be made public.

**Do you want your identity to be public for this peer review?** For information about this choice, including consent withdrawal, please see our [Privacy Policy](https://www.plos.org/privacy-policy).

Reviewer #1: No

Reviewer #2: **Yes:** Şakir Özgür Keşkek
